# Editorial: Neurofeedback: applications, techniques, and validity in clinical and non-clinical use

**DOI:** 10.3389/fnhum.2026.1960865

**Published:** 2026-09-11

**Authors:** BeomJun Min

**Affiliations:** Private Practitioner, Seoul, Republic of Korea

**Keywords:** clinical validity, EEG biomarkers, neurofeedback, self-regulation, training parameters

Neurofeedback has come a long way from its early laboratory roots. What was once a niche tool explored by a handful of researchers is now attracting serious attention across psychiatry, cognitive neuroscience, sports science, and wellness practice alike. This Research Topic was conceived to map the current state of neurofeedback across clinical and non-clinical domains, to critically evaluate the methodological standards by which its efficacy is assessed, and to identify the advances shaping its future. The four contributions gathered here approach this goal from different angles. Rather than forcing them into a single storyline, we found it more useful to let them speak to two separate, equally pressing questions the field is grappling with: how do we know neurofeedback is measuring the right thing? and once it works, why does it work—and for whom?

At its core, neurofeedback is a form of biofeedback in which individuals learn to self-regulate their own brain activity in real time, most commonly via EEG. The appeal is straightforward: rather than relying on a drug or an invasive procedure, neurofeedback invites the nervous system to reorganize itself through guided practice. This has generated particular interest for conditions such as ADHD, anxiety, and depression, as well as non-clinical applications in elite sport and stress management.

## Finding the right signal: biomarkers and the limits of a single measure

A fundamental challenge in translating electrophysiological approaches into psychiatric practice is the historical reliance on subjective clinical assessments, which often lack reproducibility. Kopańska et al. take on this problem directly, reviewing EEG and QEEG research on major depressive disorder in search of consistent biomarkers—frontal alpha asymmetry, altered theta and delta activity, and reduced gamma power. What emerges is less a settled picture than a candid one: for nearly every proposed marker, some studies report an effect in one direction and others the opposite, often depending on medication status, remission, or whether patients were assessed at rest or during an emotional task. The review's value lies in showing that QEEG's diagnostic promise is inseparable from the field's methodological fragmentation—small samples, inconsistent montages, and rarely-replicated protocols. Any future biomarker will have to survive this heterogeneity before it can be trusted at the bedside.

## Making training work: parameters, psychology, and real-world effectiveness

A second, related question is not what to measure, but how to make the training itself succeed—and here the Research Topic offers three complementary angles.

Galang et al. tackle the well-documented “neurofeedback inefficacy problem,” in which a substantial share of participants fail to learn to modulate their own brain activity. Their meta-analysis of 39 studies in healthy adults isolates concrete, actionable levers: EEG outperforms fMRI in producing measurable within-session change, complex or audiovisual feedback beats simple bar graphs, and—counterintuitively—skipping a pre-training rehearsal trial produces larger effects than including one.

Naas et al. push on one of these levers specifically: does a more aesthetically pleasing interface actually help? Their sham-controlled, 2 × 2 design disentangles the feedback experience from any genuine neural learning. The results resist a simple headline: aesthetics alone did not move motivation or perceived workload—the illusion of successfully regulating one's brain state did. Where design did matter was more conditional: pleasing visuals boosted perseverance and shifted frontal alpha activity, but mainly in older participants and specifically when the task felt difficult. “Beautiful” interfaces did not uniformly help; they helped a particular kind of user, under a particular kind of frustration.

Zooming out from single-session lab manipulations to years of clinical practice, Theis et al. report on 256 patients treated with infra-low frequency neurofeedback across four diagnostic categories. Symptom self-reports declined consistently across all groups, following the same logarithmic trajectory regardless of diagnosis, and objective performance improved in parallel. Yet whether these two kinds of improvement actually aligned diverged sharply by group: in mood and stress-related disorders, symptom relief tracked with better discrimination ability; in developmental disorders, with fewer omission errors; in childhood/adolescent behavioral disorders, there was no significant relationship at all. This is arguably the Research Topic's most important finding, and it lands on the same open question Kopańska et al. raise from the diagnostic side: an objective, physiological signal and a patient's subjective sense of improvement are not the same thing, and assuming they move together may not hold—especially in younger or more heterogeneous populations.

## Where this leaves the field

Read together, these contributions do not converge on one conclusion so much as sketch a shared frontier from different directions. Kopańska et al. remind us that even our most-studied candidate biomarker for depression remains too inconsistent to stand alone diagnostically. Galang et al. and Naas et al. show that the training experience itself—the device, the display, the sense of succeeding—shapes outcomes in ways only beginning to be mapped, and that these effects can hinge on factors as simple as age or task difficulty. Theis et al. offer the clearest evidence yet, from a large real-world cohort, that subjective relief and objective performance gain are not interchangeable, and that this decoupling itself may vary by clinical population.

Together, they argue for two priorities in future work: continued standardization of measurement and reporting, and more deliberate attention to which outcome—self-report or physiological—a given study or clinical decision actually rests on.

Neurofeedback's growing pains are not a sign that the method doesn't work; they are a sign that the field is finally asking precise enough questions to find out exactly how, and for whom, it does.

We are grateful to all authors who submitted their work to this Research Topic, to the reviewers whose expertise sharpened each manuscript, and to the editorial team at Frontiers for their support throughout the process.

